# Effects of the Applied Potential on the Performance of Polysulfone Membranes Functionalized with Sulfonated Polyether Ether Ketone Polymers

**DOI:** 10.3390/membranes13070675

**Published:** 2023-07-18

**Authors:** Abelline Fionah, Kate McLarney, Aviana Judd, Isabel C. Escobar

**Affiliations:** 1 Department of Chemistry, University of Kentucky, Lexington, KY 40506, USA; akfionah1@uky.edu; 2 Department of Materials and Chemical Engineering, University of Kentucky, Lexington, KY 40506, USA@uky.edu (K.M.); aviana.judd@uky.edu (A.J.)

**Keywords:** polyether ether ketone, sulfonation, ultrafiltration, charge, hydrophilicity, electrodialysis

## Abstract

The global water crisis growth has led to a tremendous increase in membrane technology research. Membranes are favored over many other technologies for water treatment because, in principle, they require no chemical additives and can be used isothermally at low temperatures. Membranes that can reject contaminants and salts, produce adequate permeate flux values, and require minimal cleaning are highly demanded. However, most synthesized membranes on the market have associated problems, such as membrane fouling; inverse relationships between flux and solute rejection; and the high cost of synthesis, operation, and maintenance. Therefore, there is a continuied need to produce membranes with properties that make them able to sustain flux and selectivity over time. This research study focused on increasing the surface charge and hydrophilicity of polysulfone (PSf) membranes by incorporating sulfonate-functionalized poly-ether-ether-ketone (SPEEK) into PSf/N-Methyl-2-pyrrolidone (PSf/NMP) membranes. The sulfonation of the PEEK provided a net increase in negative charge on the surface of the membranes that enabled charge repulsion to take place, thus increasing the rejection of ions. In this project, the effect of the applied potential on the performance of SPEEK: PSf/NMP membranes was evaluated. The characterization of the as-synthesized membranes was carried out using the surface’s structure and morphology, contact angle, and zeta potential. Furthermore, a voltage of 1.5 V was applied to the membranes in the presence of various salts (sodium chloride, calcium chloride, and potassium chloride salts) to evaluate the effects of the applied potential on solute rejection. It was found that both the permeability and the selectivity of the membranes increased when the voltage was applied. The obtained results indicate that incorporating SPEEK into PSf/NMP membranes increased the hydrophilicity of the membranes, and under the applied voltage, the incorporation allowed it to function as an electrodialysis process that is capable of removing ions from water bodies by utilizing the charge repulsion of ions.

## 1. Introduction

Essential practices, such as agricultural intensification [1], widespread industrialization, and urbanization [2], that are necessary to sustain civilization have led to the exhaustion of resources such as water and energy [3], leading to a global water and energy crisis [1,2,4,5]. Currently, less than 3% of the world’s water sources come from freshwater sources [6]. However, even of the available freshwater, only 1% is readily available for use, and it is unequally distributed globally, resulting in a larger percentage of the global population experiencing limited access to fresh water [7]. Various methods such as distillation, ozonation, photocatalysis, etc., have all been utilized to address this problem [5]. However, of the available methods, membrane technology using polymeric membranes has appeared to be the most prevalent due to their low energy costs and ease of use [8].

The most widely applied types of membranes in water treatment are ultrafiltration membranes (UF) [9] because of their lower pressure requirements with respect to UF membranes operation, compared to reverse osmosis (RO) and nanofiltration (NF) membranes [10] and their lower cost of production compared to microfiltration membranes(MF) [11]. However, the associated disadvantages, such as function failure in relatively high temperatures and corrosive liquids [8], high tendency of fouling, and inadequate ion rejection of ultrafiltration polymeric membranes [12], have limited their applications in membrane separations systems. Therefore, there is a need for high-performance membranes with reduced structural limitations.

Of the various types of organic membranes available in the market, such as polysulfone (PS), polyether sulfone (PES), polyacrylonitrile (PAN), and polyvinylidene fluoride (PVDF) [11,13], polysulfone-based membranes have been the most widely applied membranes in ultrafiltration [11] water treatments. This is due to their excellent properties, such as good chemical stability, good thermal stability, low resistivity, and unique mechanical properties [14,15]. However, similarly to other membrane separation technologies, polysulfone-based membranes, due to their hydrophobic nature, suffer from fouling and scaling, which limits their application in water treatment [15]. Fouling in membranes is caused by influent constituents that clog the pores of the membrane, creating a foulant layer on the membrane’s surface that cannot be easily removed by physical means such as backwashing [16]. When fouling is a result of increased inorganic ion concentrations at the membrane–water interface, this phenomenon is called scaling [17]. This can lead to the decreased permeability of the membrane, increased filtration resistance, greater energy consumption, and the need for the regular cleaning and replacement of membranes [18,19]. To reduce the effects of fouling and produce membranes that can reject salts, produce adequate permeate flux values, and require minimal cleaning, a variety of methods, which include the chemical treatment of membranes, the formation of membrane composites, surface modifications, and turning conventional membranes into electroactive membranes via the application of electrochemical membrane separation (EMS), have been found to be effective for mitigating fouling and scaling [15,19,20,21].

Poly (ether-ether-ketone) (PEEK) is a high-performance thermoplastic polymer with excellent associated thermal and mechanical properties and good proton conductivity [22]. PEEK has been applied in many industries, such as in the generation of PEM as a replacement for Nafion, in the medical field for applications as spinal implants, and in fuel cells [20,22,23]. It has a semicrystalline structure that renders it difficult to dissolve [20,24], necessitating functionalization such as the introduction of a sulfone functional group into its structure. The introduction of a sulfonic functional group to the PEEK polymer is aimed at increasing the solubility, electrophilicity, and ion exchange capacity of the polymer [20]. This is because it introduces a negative charge across the surface of the membrane. The surface modification of polymers via the introduction of charged functional groups is the most commonly applied method for the generation of charged ultrafiltration membranes [25]. Furthermore, due to the overlap of p orbitals in the π bonds, the conjugated backbone of the PEEK polymer allows the electrons to delocalize and move between atoms [26]. In the sulfonated form (SPEEK), this polymer can become conductive [27] when electrified. Electrically charged ultrafiltration membranes have been found to increase the retention of species with similar charges, hence reducing membrane fouling and increasing ion rejection. However, in addition to the traditional membrane functions of the solute’s separation via steric hindrance and charge exclusion, the electrification of such membranes can introduce further functions, such as electrochemical oxidation and reduction, electrostatic adsorption and rejection, electrophoresis, and electroporation [26].

When a dilute salt solution encounters the surface of a membrane containing a high volume of fixed charges, Donnan exclusion takes place. This is because an influx of counter ions coming into contact with the charged membrane’s surface creates an electric potential between the solution and the membrane (Donnan potential) [28,29]; i.e., the counter-ions’ concentration in the ion-exchange membrane becomes greater than that in the dilute salt solution, hence reducing the activity of the ions in salt solutions. In contrast, normally, monovalent ions such as Na^+^ and K^+^ can easily be transported across membrane surfaces; for divalent ions such as Ca^2+^, steric and dielectric exclusions and their transport across membranes are reduced [28]. However, with the introduction of a negative charge across the membrane’s surface, divalent ions can more easily be transported across the membrane via electrostatic interactions. Along with the Donnan effect, the hydration radii of chloride (Cl^−^), sodium (Na^+^), potassium (K^+^), and calcium (Ca^2+^) ions are 3.32 Å, 3.58 Å, 3.31 Å, and 4.12 Å, respectively. With Ca^2+^ having a higher charge density of 52 C mm^−3^ as a divalent cation, compared to the 8 C mm^−3^ and 11 C mm^−3^ monovalent charge densities of Na^+^ and K^+^ [30], respectively, it was hypothesized that Ca^2+^ would have a higher attraction to the negative surface of charged SPEEK membranes [29].

Although blended SPEEK membranes have shown increased stability compared to other types of blended membranes [22], there is a gap in the literature focusing on their ability for charge repulsion in the absence and presence of the applied voltage for water separations applications. The purpose of this research study was to increase the surface charge and hydrophilicity of polysulfone (PSf) ultrafiltration (UF) membranes by blending a high-performance sulfonate-functionalized poly-ether-ether-ketone (SPEEK) polymer into PSf/N-Methyl-2-pyrrolidone (PSf/NMP) membranes. The membrane composite’s evolution was first evaluated in the presence of 0%, 5%, and 10% weight percentage SPEEK incorporation. Then, the membrane’s performance was evaluated in terms of evaporation time, permeability, and rejection. Finally, the ability of the blended membrane to remove charged salts, namely sodium chloride, calcium chloride, and potassium chloride, from water in both the presence and absence of an applied potential difference via charge repulsion was investigated.

## 2. Materials and Methods

### 2.1. Chemicals

N-Methyl-2-pyrrolidone (NMP), potassium chloride (KCl), sodium chloride (NaCl), calcium chloride (CaCl), sodium hydroxide (NaOH) salts, fuming sulfuric acid, bovine serum albumin (BSA), and ACS-grade hydrochloric acid (HCl) were obtained from VWR international (Solon, OH, USA). Polysulfone (PSf) and cellulose acetate at molecular weights (mw) of 40,000 and 100,000 g/mol were obtained from Sigma Aldrich (St. Louis, MI, USA). Polyether ether ketone (PEEK) was obtained from Polysciences Inc. (Warrington, PA, USA). Polyethylene glycol (PEG) at molecular weights of 200, 600, 1000, 4000, 8000, 10,000, and 20,000 g/mol was obtained from Alfa Aesar (Ward Hill, MA, USA).

### 2.2. Experimental

#### 2.2.1. Sulfonation of SPEEK

The sulfonation process was adapted from previous studies [31]. In summary, PEEK was dissolved at room temperature in a 95:5% ratio concentrated sulfuric acid/fuming sulfuric acid solution. Sulfonated PEEK was precipitated out of the solution. The precipitant was neutralized and dried overnight in a vacuum oven at 60 °C. At room temperature and in concentrated sulfuric acid, one sulfone functional group per unit can be selectively introduced into the PEEK backbone. This makes sulfonation especially suitable for aromatic polymers such as PEEK [32]. Furthermore, the sulfonation of PEEK in concentrated H_2_SO_4_ has been reported to be free of degradation and cross-linking reactions [33], producing purer results. The arrow-pushing mechanism can be seen in Figure S1.

#### 2.2.2. Membrane Polymer Composite Evolution: 0% to 5% to 10% SPEEK

The membranes were formed via the method of non-solvent-induced phase separation, as previously reported [31]. The non-solvent utilized was DI water. The dope solution was prepared by dissolving polysulfone (PSf) blended with SPEEK in a 95/5% ratio into NMP solvent (83:17% solvent to polymer ratio) at room temperature. The dope solution was degassed in an Elma Schmidbauer GmbH (Singen, Germany) sonicator at 25 °C for 60 min to remove air bubbles. The membranes were prepared via the nonsolvent induce phase inversion separation method (NIPS). This was essential to maintain greater control of the interaction between the polymer and the solvent [34]. The dope solution was then poured onto a glass plate and spread/cast using a doctor blade (Paul N Gardner, Pompano Beach, FL, USA). The film on the glass plate was then immersed in water at room temperature. Control dope solutions (solutions without SPEEK polymer) were prepared at an 83:17% NMP:PSf ratio, and the membrane was formed in a similar manner. The membrane composition for each membrane can be observed in Table 1.

### 2.3. Characterization

#### 2.3.1. Cloud Point Measurements

The cloud point procedure was adapted from the literature [35]. Here, two approaches were utilized in the making of the dope solutions for the cloud points. In the first one, the total polymer and SPEEK percentages added were maintained constant. In this step, control solutions of 5, 10, 15, and 20% PSf were made by combining PSf and NMP at room temperature and mixing until the solution was homogenous. Using the same procedure, solutions of the same polymer percentage (5, 10, 15, and 20% polymer) were made with a constant PSf: SPEEK weight percentage ratio of 95:5, respectively. This was repeated when the SPEEK percentage was increased to 10%. In the second approach, the percentage of PSf was maintained constant at 17%, and varying percentages of SPEEK were added to the matrix. For the cloud point, 5 mL of homogenous solution was heated to 60 °C and stirred constantly. Local precipitation was observed when water was dropwise added to the solution [36]. The solution was heated and constantly stirred so local precipitation would dissolve more quickly and cloud points could be reached more efficiently. Ultra-pure deionized water was added to the solution 30 μL at a time once local precipitation from previous additions had dissolved. The cloud point was reached when the solution was visibly cloudy throughout, rather than precipitation being localized in one region. Weight fractions were calculated from the average volume of water added to the solution.

#### 2.3.2. Structural Polymer Evolution

##### Fourier Transform Infrared Spectroscopy (FT-IR)

To further understand the functionality of the as-synthesized membranes, FT-IR measurements were carried out using a NEXUS 470/670/870, 110 W, 5 V/12 V ESD (Thermo Nicolet, Madison, WI, USA). Membranes were dried overnight prior to obtaining the spectra, and various spectra of the membranes were obtained and averaged.

##### X-ray Photoelectron Spectroscopy (XPS) and Depth Profile

The elemental composition of the as-synthesized membranes as well as the depth profile was carried out using a Thermo Scientific K-Alpha XPS (Waltham, MA, USA). Atomic percentages of the various chemical compounds on the surface of the membranes were obtained. The depth profile was carried out to determine whether the presence of the SO_3_- functionalization was maintained on the surface or within the pore’s structure. For the depth profile, the membranes were etched 20 times for over 1000 sec each.

##### NMR Analysis

The confirmation of SPEEK integration into the PSf/NMP membrane matrix was performed using 1HNMR Spectroscopy in a deuterated chloroform solvent. The proton NMR (1H-NMR) spectra of the membrane samples were obtained using Bruker NMR (Billerica, MA, USA) at a frequency of 400 MHz. The assignment of peak signals was obtained from the literature [37].

#### 2.3.3. Morphological Polymer Evolution

##### Scanning Electron Microscopy (SEM)

The surface and cross-sectional morphologies of the as-synthesized membranes were investigated using the FEI Quanta 250 scanning electron microscopy (SEM, ThermoFisher Scientific, Hillsboro, OR, USA). For cross-sectional morphologies, cryo-snap/rapture was utilized to reduce the effects of distortion, improving the resolution of the resulting image at the cut surfaces [38]. Liquid nitrogen was used for the purposes of cryo-snapping.

##### Pore Size

**Figure 1:**
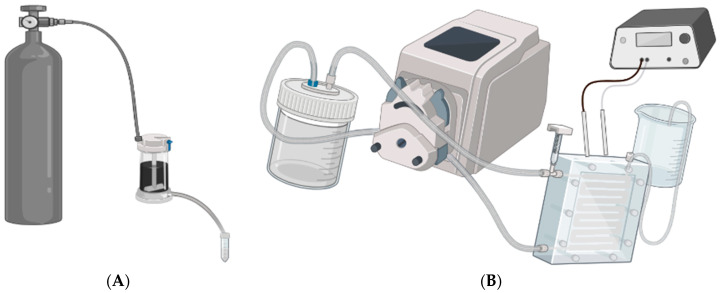
Schematic diagrams showing the experimental setup for the dead-end filtration system in (**A**) and the crossflow filtration system in (**B**) as used in the performance analysis of the as-synthesized membranes in this study.

The pore size of the membranes was determined using the molecular weight cut-off (MWCO) method, and SEM image analyses were carried out using ImageJ [39]. Using a Steritech crossflow filtration cell with a maximum operating pressure of 41.4 bar (Figure 1A), the membranes were pre-compacted. The pre-compaction of membranes was carried out to flush out any impurities, such as any possible leftover solvent, that may have been trapped in the pores of the membranes [40]. Then, 200 g/mol of PEG MW at a concentration of 200 ppm was filtered through the membranes for 15 min. Samples were collected and then analyzed for TOC. The MWCO was related to the radius of the pores via Equation (1) [41]:

(1) $$rH=0.06127{\left(MW\right)}^{0.3931}$$

where *MW* is the molecular weight (KDa), and *rH* is the hydrodynamic radius (nm). This was repeated for the PEG molecular weights of 600, 1000, 4000, 8000, 10,000, and 20,000 g/mol and 40,000 mw; and cellulose acetate at mw 40,000 and 100,000. These materials were chosen due to their minimal interaction with the membrane’s material [42].

#### 2.3.4. Membrane Functional Properties

##### Zeta Potential

The zeta potential was obtained using an Anton Paar SurPASS zeta instrument (Ashland, VA, USA) to determine the surface charge of the membrane [43]. All solutions were made in Type I DI water [44]. The analyte solution contained a 0.1 M KCl solution. The pH of the solution was adjusted using HCl and NaOH solutions, with both at 0.5 M. The streaming current was obtained and averaged per sample for use in calculating the zeta potential.

##### Membrane Wettability

To evaluate the hydrophilicity and hydrophobicity of the synthesized membranes, water contact angle measurements were obtained using a drop shape analyzer (Kruss DSA100, Matthews, NC, USA). The membranes were air-dried overnight before measurements were obtained using the sessile drop method, and 6 measurements obtained at various surface points were averaged to obtain a net contact angle measurement.

##### Percent Water Uptake

To further understand the hydrophilic nature [45] of the SPEEK-embedded membranes and deduce the number of exchangeable ionic groups present per dry membrane weight [46], water uptake and ion exchange capacity (IEC) measurements were carried out, respectively. The procedures for water uptake and ion exchange capacity were obtained from the literature [47] and adapted for the purposes of this study. For water uptake, the membranes were dried in a vacuum oven at 40 °C for 48 h and the dry weight was obtained. Then, the dry membranes were submerged in 100 mL of deionized water at room temperature for 48 h to obtain their wet weight by taking careful consideration to remove excess water. The water uptake (*WU*, wt.%) was then calculated. The water uptake (*WU*, wt.%) was then calculated according to Equation (2) [48]:

(2) $$WU=\left(\left(Ws-Wd\right)/Wd\right)\times 100\%$$

where *WU* is the water uptake, *Ws* is the weight of the wet membranes, and *Wd* is the weight of the dry membranes.

##### Ion Exchange Capacity

For ion exchange capacity measurements, the pre-weighted dry membranes were soaked in a 0.1 M HCl solution for 48 h to allow the maximum acidification of membranes. Then, the membranes were removed from the acid solution, thoroughly washed with DI water, and soaked for an additional 48 h in a 2 M NaCl solution to allow proton exchange to take place. The NaCl solution was then titrated against a 4 mM NaOH solution with phenolphthalein as the indicator, and the IEC was calculated. The calculation of the IEC was carried out with the consideration of the neutralization point at which a PH of 7 was reached and the point when the 1st color change was observed. The IEC was calculated according to Equation (3) and the degree of sulfonation (DS) was deducted from the IEC equation according to Equation (4) [49]

(3) $$IEC=M\left(NaOH\right)*\;V\left(NaOH\right)/Wd)$$

(4) $$DS\left(\%\right)=M\left(p\right)*IEC1000-\left(IEC*M\left(f\right)\right)\times 100$$

### 2.4. Performance Analysis

Filtration experiments were carried out in both a dead filtration cell at constant pressure and a cross-flow filtration cell at varying pressures. In both filtration tests, the pre-compaction of the membrane was carried out using water, followed by the filtration of salt solutions and then reverse flow filtrations (backwashing or hydraulic back flush), which were carried out to determine the potential for membrane reuse. In membrane filtration, it is utilized to loosen the foulant cake layer on membrane surfaces [50]. The reverse flow was performed by flipping the membranes over in filtration cells and filtering water through the membrane to determine the membrane’s regeneration potential [51] and establish whether the interacting ion between the contaminant and the charged membrane surface was irreversible.

#### 2.4.1. Constant Pressure Filtration

A Millipore^®^ dead-end cell (Burlington, MA, USA) with a maximum pressure allowance of 5.17 bar (75 psi) was used (Figure 1B). At a constant pressure of 4.14 bar (60 psi), the membranes were pre-compacted. Then, seven samples of feed solutions (Table 2) were filtered through the cells, and the periods of time taken for filtration were recorded. The solutions consisted of NaCl, KCl, and CaCl_2_ of 1 mM and 5 mM concentrations as well as BSA proteins at 100 ppm concentrations. The collected samples were analyzed for both flux and rejection.

#### 2.4.2. Constant Flow Filtration

A Steritech cross-flow filtration cell with a maximum operating pressure of 6.9 bar (100 psi) was used, and pressure was allowed to vary across filtration trials (Figure 1A). The pre-compaction of membranes was carried out using DI water. Ten samples of feed solutions were taken using salt solutions, as shown in Table 2. Then, the voltage was applied in the forward/first E-field across the membrane, and ten samples were collected. Reverse voltage/second E-field was applied across the membrane, and ten samples were collected. The membrane was washed and flipped over for the collection of reverse flow samples. This was repeated for all salts (NaCl, KCl, and CaCl_2_), at concentrations of 1 mM and 5 mM and a voltage of 1.5 V.

## 3. Results

### 3.1. Cloud Point Analysis

**Figure 2:**
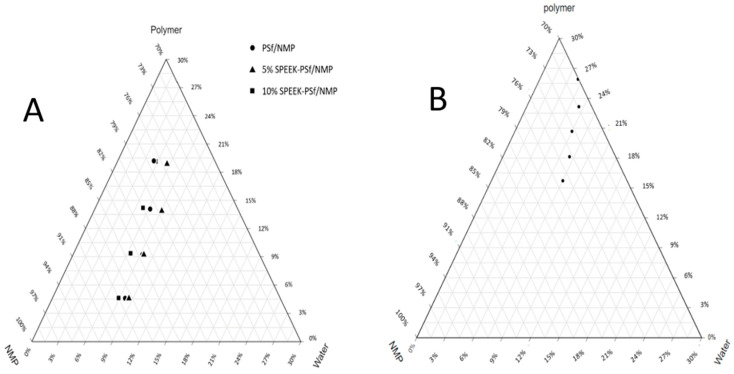
Ternary phase diagrams showing the cloud point measurements of dope solutions containing PSf/NMP, 5% SPEEK-PSf/NMP, and 10%SPEEK-PSf/NMP. In (**A**), the total polymer contribution was maintained at 17%, while in (**B**), the percentage of incorporated PSf was held constant.

Cloud point analysis can be used to determine the point at which a liquid–solid phase change occurs. For application with non-solvent-induced phase inversion membranes, the cloud point is significant as an indicator for the beginning of membrane formation [52]. The objective of this cloud point analysis was to evaluate the thermodynamic compatibility of SPEEK and PSf polymers, to determine the driving polymer (SPEEK or PSf) for membrane formation, and to compare the point of phase change for the dope PSf/NMP and SPEEK-PSf/NMP solutions. Although both polymers have great mechanical and physical properties, PSf’s functional unit is larger than that of SPEEK. Cloud point analysis, observed in the isothermal ternary phase diagram (Figure 2A), demonstrated that for dope solutions with a low-weight-percentage polymer, the liquid–solid phase change for PSf/NMP and SPEEK-PSf/NMP solutions occurred at roughly the same weight% of water. For 15 and 20 weight% polymers, the cloud points of 5% SPEEK-PSf/NMP dope solutions were observed at a higher weight% of water than both the PSf/NMP and 10%SPEEK-PSf/NMP dope solutions. This indicated that adding the 5% SPEEK polymer to the dope solution not only increased the overall miscibility of the solution but also reduced the precipitation rate. The shifting of the binodal lines away from the polymer solvent axis indicated that 5% SPEEK-PSf/NMP membranes required more nonsolvent (water) to form. The results for the PSf/NMP dope solution were comparable to those obtained by Ismail et al. However, their study was carried out at 25 °C [35]. This means that regardless of the temperature at which the cloud point measurements were obtained, a similar trend should be observed. In Figure 2B, the weight % of PSf was maintained at 17%, and various weight percentages ranging from 0 to 10% of the SPEEK polymer were added to form the dope solutions. Here, it was observed that as the total polymer weight percentage increased, the binodal lines shifted more towards the spinodal line, indicating that there was an introduction of local phase instability where demixing was instantaneous [53]. This could lead to the formation of large finger-like microvoids in the membrane’s pore structure [53,54]. Furthermore, the results in Figure 2B indicated that not only was more non-solvent required for phase transition to occur, but SPEEK was also the main polymer influencing the rate of membrane formation in the composite.

### 3.2. Membrane Characterization

#### 3.2.1. Structural Polymer Evolution

##### Fourier Transform Infrared Spectroscopy (FT-IR)

**Figure 3:**
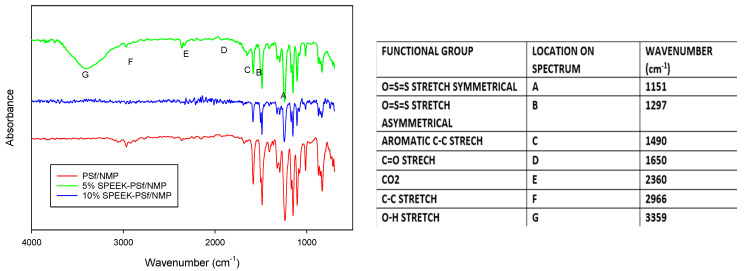
FTIR spectra showing the functional peaks within PSf/NMP, 5% SPEEK−PSf/NMP, and 10% SPEEK−PSf/NMP membranes.

The FTIR spectra of a PSf/NMP membrane and both 5% and 10% SPEEK-PSf/NMP composite membranes are shown in Figure 3. The strong absorption peaks around 1490, 1151, and 1297 cm^−1^ correspond to the aromatic backbone and symmetric O=S=O and asymmetrical O=S=O stretching, respectively, that can be observed in both PSf and SPEEK. The vibration of the carbonyl functional group C=O was observed at 1650 cm^−1^ [55]. This differs from the 1750 cm^−1^ often observed in the literature due to shifting associated with conjugation between the aromatic ring and the phenolic oxygen observed in the backbone of both PSf and SPEEK [56]. In contrast to the PSf/NMP membrane, the 5% SPEEK-PSf/NMP membrane exhibited a broad band in the high energy region around 3359 cm^−1^ corresponding to the OH functional group [55] of the SO_3_H group associated with the incorporation of SPEEK into the membrane matrix. The same peak should be observed in 10% SPEEK-PSf/NMP membranes; however, the spectrum seemed identical to that of PSf/NMP membranes. This was hypothesized to potentially be a result of the following: while the SO_3_H functional groups may have been introduced into the membrane matrix, they may not have been localized on the surface.

##### X-ray Photoelectron Spectroscopy (XPS) and Depth Profile

XPS analysis (Table 3) showed the presence of S, C, and O compounds in both PSf/NMP and SPEEK-PSf/NMP membranes. The percentage of O1s was reduced from 22.35% to 15.38%. The reduced percentage of O1s in 5% SPEEK-PSf/NMP compared to the control (PSf/NMP) could be attributed to the composite’s formation where there was a decrease in the O=S=O functional group or the formation of new carbon–oxygen bonds [31]. Here, 5% S PEEK-PSf/NMP exhibited a peak at 167.8, which is characteristic of the SO_3_H functional group [57]. Consequentially, the percentage of S2p characters increased with the introduction of 5% SPEEK into the membrane matrix from 1.95% to 3.01%. This showed that the SPEEK polymer was incorporated into the membrane. This was further supported by the EDS spectra observed in Figure S2. Increasing the percent SPEEK incorporated from 5% to 10% did not double the S2p content, as expected. All atomic % of all atoms of interest was similar to those of PSf/NMP membranes, with a slight increase in C1s from 75.71 to 76.66% and in S2p contents from 1.95% to 2.18%. There was also a slight decrease in O1s content from 22.35% to 21.15%, which again could be attributed to the decrease in the O=S=O functional group. XPS results showed that even though there was a change in the structural elemental composition of PSf/NMP membranes when SPEEK was introduced into the matrix, the difference was more pronounced when 5% SPEEK was incorporated as opposed to 10% SPEEK, which agrees with FTIR spectra (Figure 3).

**Figure 4:**
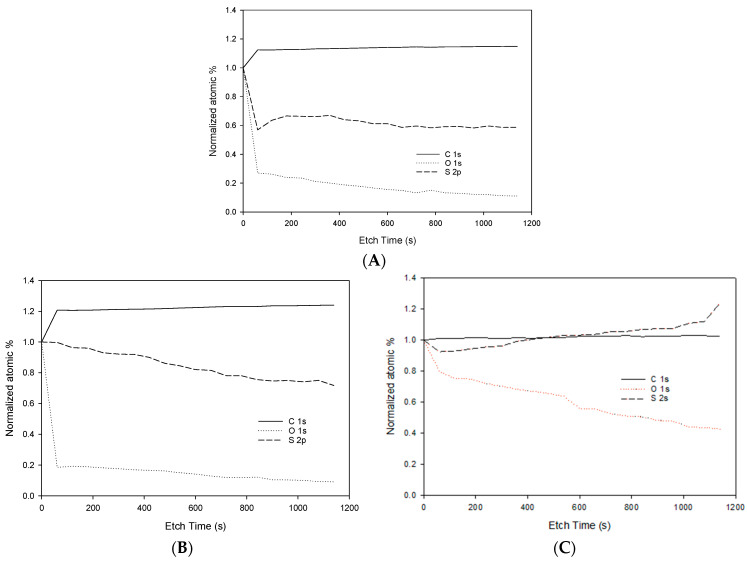
XPS depth profile spectra for membranes PSf/NMP in (**A**), 5% SPEEK-PSf/NMP in (**B**), and 10% SPEEK-PSf/NMP in (**C**). Each membrane was etched for over 1100 seconds, and points were taken after each level of etching.

To further show that the surface of the PSf/NMP membrane was functionalized with SPEEK as opposed to the pores in the 5% SPEEK-PSf/NMP membranes, XPS depth analysis was carried out. Figure 4 shows the normalized atomic percentages of C1s, S2p, and O1s for both the functionalized membrane (5% SPEEK-PSf/NMP) and the unfunctionalized membrane (PSf/NMP). Starting at an etch time of 0s, C1s’ atomic percentage increased for all membranes: PSf/NMP in Figure 4A, 5% SPEEK-PSf/NMP in Figure 4B, and 10% SPEEK-PSf/NMP in Figure 4C. There was increased C1s content in the membrane that contained 5% SPEEK-PSf/NMP compared to those of both PSf/NMP and 10% SPEEK-PSf/NMP. This could be attributed to the introduction of increased aromatic rings that are prevalent in the PEEK backbone. With respect to 10% SPEEK-PSf/NMP membranes, the C1s’ atomic percentage remained the same as the membrane was etched. This was similar to what was obtained in the PSf/NMP membrane. Since this was similar to the membranes in which no SPEEK was incorporated, it could indicate that there was a limited incorporation of 10% into the PSf/NMP membrane matrix. Subsequentially, O1s and S2p contents were reduced significantly in both PSf/NMP and 5% SPEEK-PSf/NMP membranes. The reduction in S2p content indicated that SPEEK was localized more on the membrane’s surface than the pores. S2p content was reduced less in the SPEEK-PSf/NMP membrane compared to that of the PSf/NMP membrane. This indicated that although much of the functionalization took place at the surface of the membrane, there was still slight leaching into the pore’s structure. These results were similar to those obtained in the literature [58]. In 10% SPEEK-PSf/NMP membranes, the S2p content showed a slight decline at the first few etch levels but ultimately increased further in the pores of the membrane that was etched. This indicated that, potentially, the 10% SPEEK was incorporated more in the pore’s structure rather than on the surface.

##### NMR Analysis

**Figure 5:**
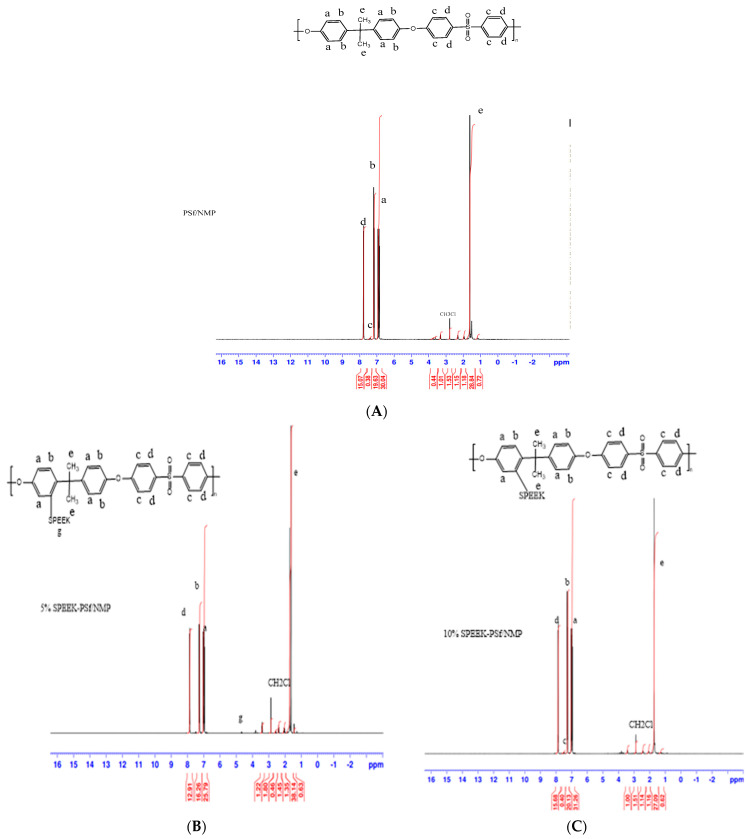
NMR spectra of (**A**) PSf/NMP, (**B**) 5% SPEEK-PSf/NMP, and (**C**) 10% SPEEK-PSf/NMP membranes prepared in deuterated chloroform.

The introduction of the SPEEK polymer into the membrane composite was confirmed using liquid-phase NMR analysis. This was aimed at investigating whether SPEEK was being integrated into the polymer backbone. In Figure 5B, for 5% SPEEK-PSf/NMP membranes, a new peak signal at 4.75 ppm shows the introduction of the SPEEK polymer. The reduction in/disappearance of peak b also indicates that the H signal was substituted for SPEEK. There was no considerable difference between 10% SPEEK-PSf/NMP and the control PSf/NMP membranes, as observed in Figure 5C and A, respectively. This can be attributed to SPEEK being incorporated into the polymer backbone or perhaps simply having been leached out. These results can be compared to the FTIR in Figure 3 where the OH band was prevalent in 5% SPEEK but was absent in 10% SPEEK These results are expected to correlate with the surface charge, with 5% SPEEK showing a higher potential for a negative charge across the membrane’s surface as opposed to both the control PSf/NMP and 10% SPEEK-PSf/NMP membranes.

#### 3.2.2. Morphological Polymer Evolution

##### Scanning Electron Microscopy (SEM)

**Figure 6:**
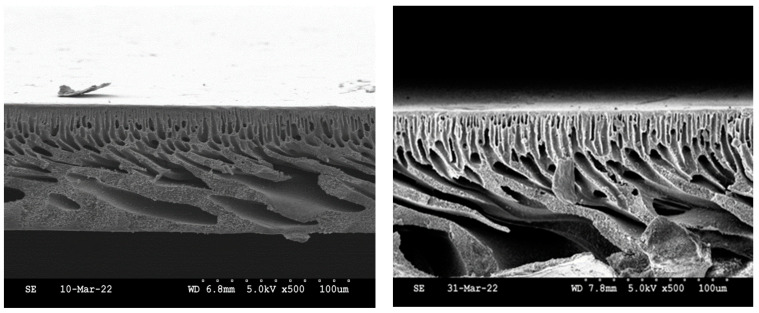
Cross-sectional SEM images of PSf/NMP (**left**) and SPEEK-PSF/NMP (**right**) membranes. Cry rapture was utilized to obtain cross-sectional images.

SEM images obtained for both SPEEK-PSf/NMP membranes and PSf/NMP membranes, as shown in Figure 6, showed finger-like projections for pore structures. The polymer-rich active layer at the top of the membranes is more pronounced in SPEEK-PSf/NMP membranes as opposed to the PSf/NMP membrane. During membrane formation via the NIPS process, when the thin film is immersed into the nonsolvent, the rate of mixing and de-mixing plays an important factor in the types of pores formed. When the de-mixing rate is very fast, larger microvoids form [53]. Since the ternary phase diagrams (Figure 2A) show that dope solutions with 5% SPEEK incorporated into them were more miscible and closer to the spinodal lines, it should be expected that the finger-like microvoids formed should be larger. From the SEM images, there were more pore projections in the SPEEK-PSf/NMP membranes, in agreement with the ternary phase diagrams. This suggested that the membrane could have higher permeability/flux [53,59].

##### Pore Size

**Figure 7:**
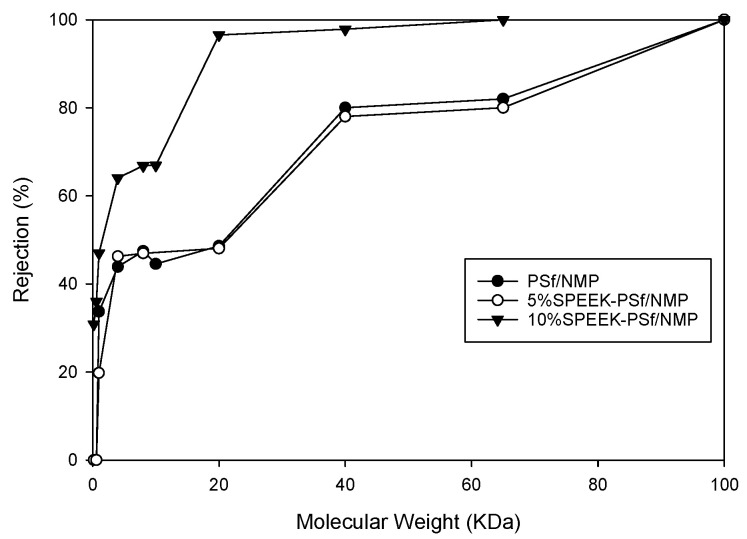
MWCO of PSf/NMP, 5% SPEEK-PSf/NMP, and 10% SPEEK-PSf/NMP membranes obtained from filtering PEG solutions at various molecular weights and analyzed by the TOC analyzer.

The average pore size of the 5% SPEEK-PSf/NMP membrane was measured to be approximately 0.05+/−0.024 microns, which was larger than that of the PSF/NMP membrane (0.04+/−0.012 microns). This agreed with the surface images obtained from SEM where the pores appeared to be larger in the composite membrane than the control. This was further reinforced by the MWCO measurements (molecular weight at which 90% of the macromolecular solute is rejected by the membrane [60]). From MWCO measurements, as observed in Figure 7, both SPEEK-PSf/NMP membranes and PSf/NMP membranes were within the ultrafiltration range. Increasing the percentage of SPEEK incorporated into the membrane matrix from 5% to 10% reduced the MWCO of membranes. For a membrane in the UF/NF range, an increase in MWCO can lead to an increase in permeability and a decrease in percentage rejection and vice versa [61].

#### 3.2.3. Membrane Functional Properties

##### Zeta Potential/Surface Charge

**Figure 8:**
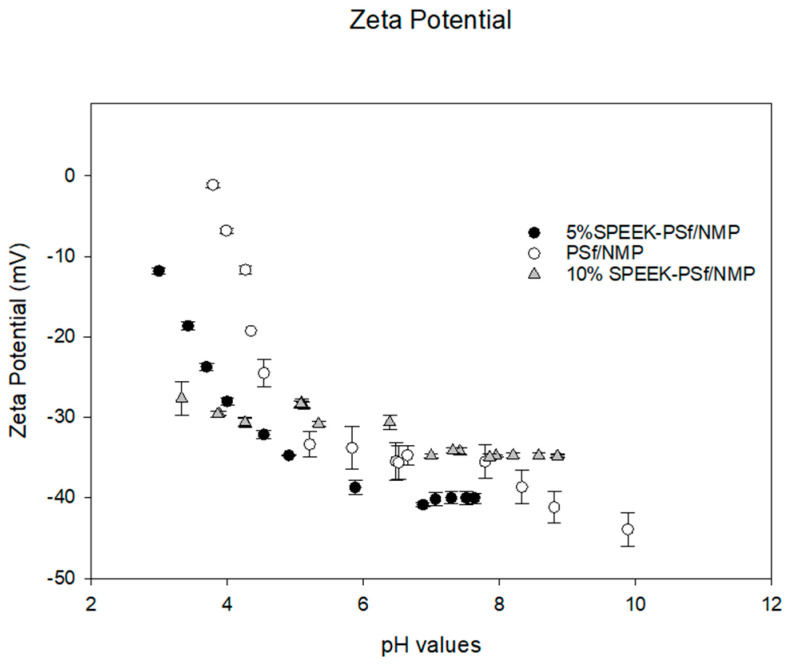
Zeta potential for PSf/NMP, 5% SPEEK-PSf/NMP, and 10% SPEEK-PSf/NMP membranes over a range of pH values. NaOH and HCL were utilized for pH adjustments.

For membranes to be competitive, they are often required to be solvent resistant, durable, and have a long shelf life. This leads to the use of polymers with higher hydrophobicity properties that are prone to fouling and, hence, decreased membrane performance [27]. By increasing the sulfonation and hydrophilicity, as shown in Figure 8, the charge increases, hence improving its performance [62]. Figure 8 shows the pH dependence of the membrane’s zeta potential. Here, 5% SPEEK-PSf/NMP membranes illustrated an overall more negative charge across all pH values that were evaluated. This showed that the introduction of just the 5% SPEEK polymer into the membrane matrix greatly influenced the charge distribution of the membrane. The addition of a higher percentage of the SPEEK polymer should theoretically also further increase the zeta potential; however, upon investigation, it was observed that at lower pH values of 3 to 4, the membrane with 10% SPEEK was more negatively charged, and the charge became more positive than that of 5% SPEEK as the pH increased. This could be attributed to the fact that more of the charge was introduced within the membrane pores rather than only on the surface, as indicated by the depth profile in Figure 4.

##### Wettability

**Figure 9:**
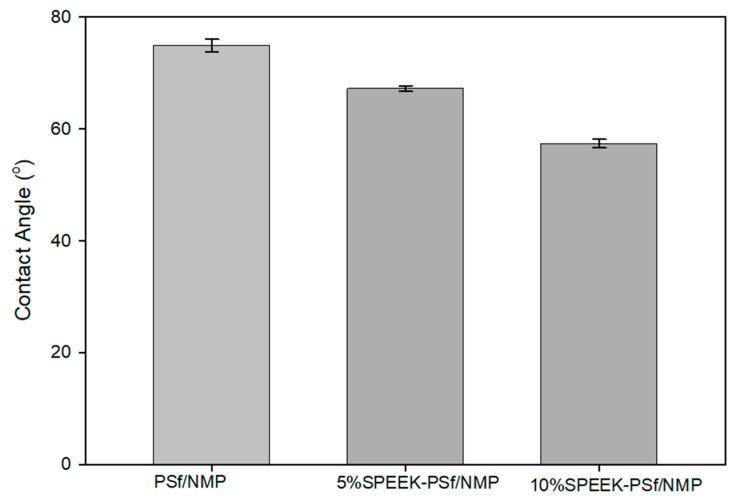
Contact angle measurements of PSf/NMP, 5% SPEEK-PSf/NMP membranes, and 10% SPEEK-PSf/NMP membranes obtained using the sessile drop method.

The hydrophilicity of the membrane is based on the intermolecular interactions between the surface of the synthesized membranes and a droplet of water. A hydrogen-terminated compound will produce a higher contact angle of 90 degrees and above [63]. However, as more functional groups are introduced into the surface of the compounds, the contact angles of the surfaces of such membranes can decrease or increase depending on the type of functional group introduced [64]. Due to the inverse relationship between contact angle measurements and hydrophilicity, this would deem a compound with lower contact angle measurements more hydrophilic. From the FT-IR in Figure 3, the PSf/NMP membrane fosters a ketone functional group. The presence of oxygen functionality reduced the contact angle of the membrane and made it slightly less hydrophobic, giving it a contact angle measurement of 75 ± 0.5°. The main reason for incorporating SPEEK into the PSf/NMP membrane was to increase the hydrophilicity of the membrane. PEEK reduces its pKa value to 1. This has been reported to be much smaller than that of other membranes on the market such as Nafion, which has a pKa of 6. This indicates that SPEEK can have higher conductivity under similar conditions. The sulfone functional group that was introduced into the membrane matrix increased the amount of oxygen within the compound. This further reduced the contact angle to 67 ± 1.1°, indicating that the membrane had become more hydrophilic in nature, as observed in Figure 9, increasing the amount of SPEEK from 5% to 10% and further reducing the contact angle measurements to 57.4 ± 0.7°. This agrees with the literature: At a high enough percentage, SPEEK can dissolve in water, implying its higher hydrophilicity [33]. While it may seem that the contact angle contradicted the results obtained via FT-IR in Figure 3 and the NMR analysis in Figure 5, it is hypothesized that the majority of SPEEK was concentrated within the pores rather than the surface when 10% was added. This hypothesis was supported by XPS depth profiling, as shown in Figure 4, which shows that S2p content increased further into the pores belonging to the etched membrane.

##### Water Uptake and Ion Exchange Capacity (IEC)

**Figure 10:**
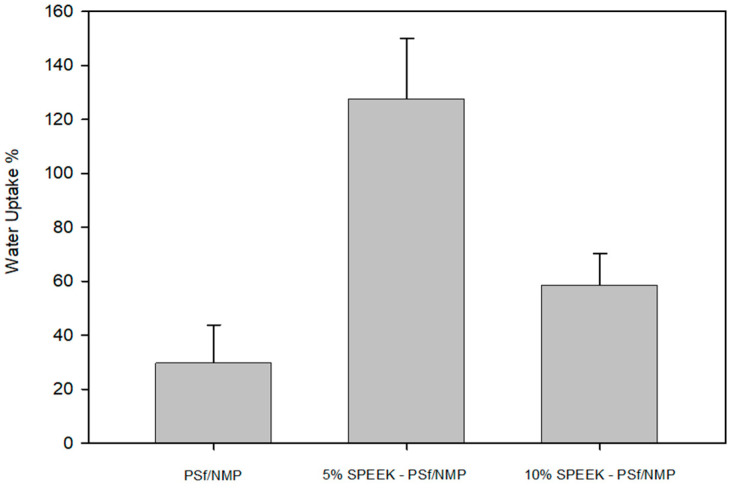
Percentage of water uptake of PSf/NMP, 5% SPEEK-PSf/NMP, and 10% SPEEK-PSf/NMP membranes.

The ability of a membrane to selectively allow the passage of cations while providing a barrier to anion passage has been associated with the electrodialysis water transport mechanism [65]. The percentage of water uptake provides information on how the counter-ion is transported through the hydrophilic region of the membrane. In the case of the membranes described within this study, this provides information on the effect of sodium transport across the polymer surface and the effects of electrostatic interactions with the negatively charged groups [42]. From Figure 10, 5% SPEEK-PSf/NMP membranes had the highest percentage of water uptake, which was more than three times that of the PSf/NMP membranes and twice as much as that of the 10% SPEEK-PSf/NMP membranes. The increase in the percentage of water uptake capacity indicated that the incorporation of SPEEK into the PSf/NMP membrane matrix led to the faster transport of the Na ion across the membrane surface.

##### Ion Exchange Capacity (IEC)

**Figure 11:**
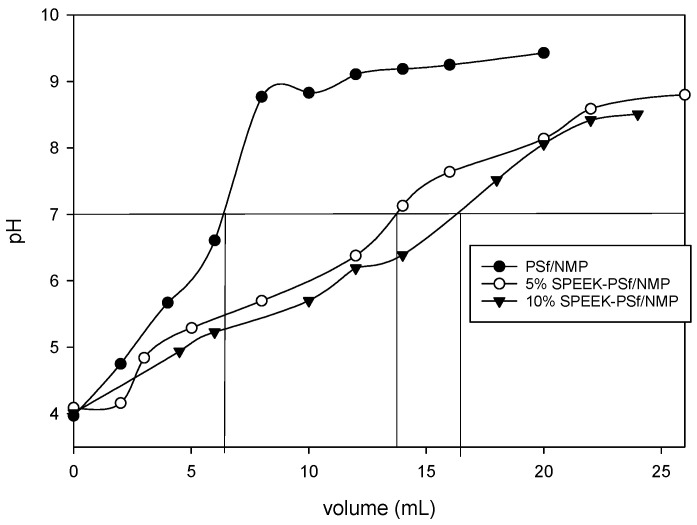
Volume of water needed to reach the equilibrium for the calculation of IEC for PSf/NMP, 5% SPEEK-PSf/NMP, and 10% SPEEK-PSf/NMP membranes.

Measuring ion-exchange capacity (IEC) permits the determination of the total functional groups responsible for ion exchange in polymer electrolyte membranes based on weight [65]. An increase in membrane IECs has been correlated with improved membrane performance with respect to permeability and increased membrane swelling [32,33]. The results summarized in Table 4 show the IEC calculated from the point at which a color change was observed, and it was observed to be the highest for 5% SPEEK-PSf/NMP at 0.608485 and the lowest for the PSf/NMP membranes at 0.202286. These results agreed with previous results, such as the zeta potential (Figure 8), NMR (Figure 9), and FTIR (Figure 5), which all indicated that the 5% SPEEK-PSf/NMP membranes had more negatively charged functional groups available on the surface compared to the 10% SPEEK-PSf/NMP and PSf/NMP membranes. These data were utilized to calculate the degree of sulfonation within the membranes via Equation (4), and it was observed (Table 4) that the SD was highest for membranes made of 5% SPEEK-PSf/NMP at 0.1842 compared to 10% SPEEK-PSf/NMP and PSf/NMP membranes at 0.1247 and 0.05922, respectively. It was however observed in Figure 11 and Figure S3 that it took more base to completely neutralize the 10% SPEEK-PSf/NMP membranes than either the 5% SPEEK-PSf/NMP or PSf/NMP membranes. This was contradictory to what was observed from other characterization methods. A higher weight percentage of SPEEK has been associated with hydrogel formation. This means that for specific 10% SPEEK-PSf/NMP membranes, there could have been some polymer agglomeration that was concentrated in the specified regions of the membranes [32]. This would still require more base to neutralize it without increasing the overall sulfonation degree of the membrane. Another possible explanation could be that SPEEK could have been behaving as a filler in the membrane matrix, and its percolation threshold had been effectively realized by 5% SPEEK [32]. Because of this, a higher percentage of SPEEK would in turn form clusters in the matrix and effectively diffuse out of the membrane during the mixing and de-mixing process in the phase transition.

From structural and morphological analyses, it was determined that the sulfonation of the PEEK polymer could introduce a negative sulfone functional group onto the surface of the membrane and integrating the sulfonated PEEK polymer into PSf/NMP membranes effectively increased their hydrophilicity and the net negative charge on the membrane’s surfaces. Increasing the percentage of the incorporated SPEEK from 5% to 10% did not improve the functional properties of the polymeric membrane blend. Therefore, 5% SPEEK-PSf/NMP membranes were chosen for permeability studies.

### 3.3. Filtration Studies

#### 3.3.1. Effect of Solvent Evaporation Time on the Performance of the Membrane

**Figure 12:**
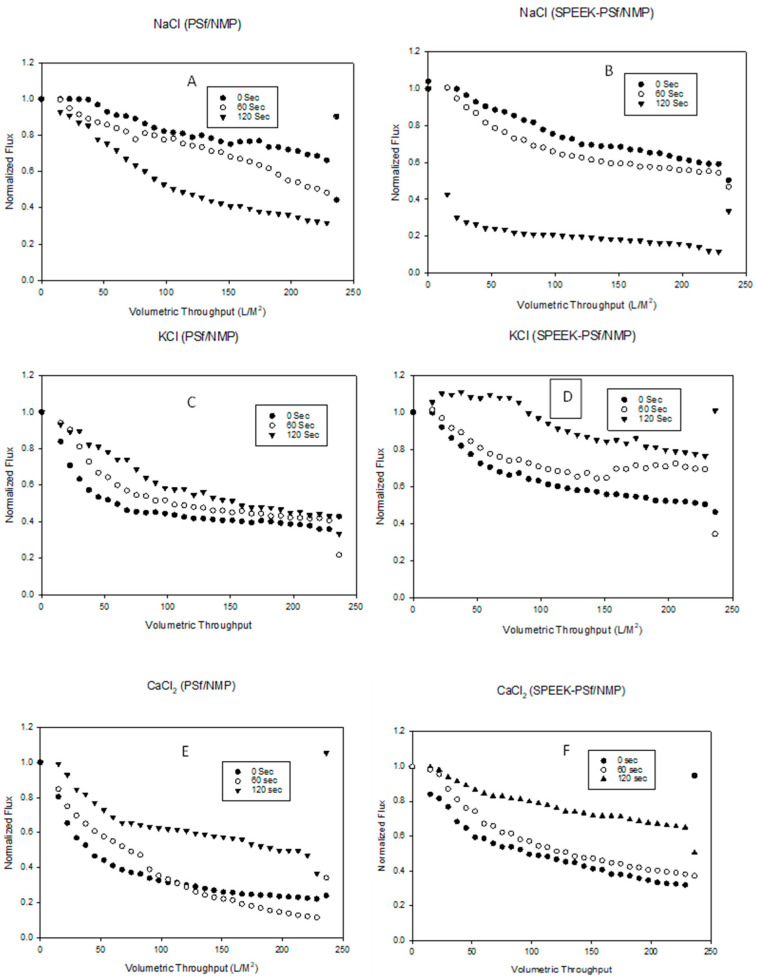
Graphs showing the effects of evaporation time on the retention of salt species NaCl (**A**,**B**), KCl (**C**,**D**), and CaCl_2_ (**E**,**F**) on both PSf/NMP (**left**) and 5% SPEEK-PSf/NMP (**right**) membranes. The first 10 points are from the pre-compaction step. The next 10 points show the salt filtration at 1 mM salt concentration, and the third set of 10 points shows the 5 mM salt concentration filtrations. The final one point shows the backwashing step and concludes the filtration cycle.

The solvent evaporation time plays a crucial role in membrane performance. The selectiveness of a membrane can be tuned by increasing or reducing the evaporation time. It has been determined that, during NIPS, the longer the evaporation time before immersion into the non-solvent, the thicker the polymer-rich top layer of the surface [66,67]. This can reduce the flux of the membranes while increasing rejection. From the XPS depth profile spectra (Figure 4B), it was concluded that the negative charge was concentrated on the surface of 5% SPEEK-PSf/NMP membranes rather than within the pores. From this, it was hypothesized that a longer evaporation time should produce a thicker active layer (polymer-rich layer) with more negative charges accessible for use in the charge repulsion of negatively charged contaminants. The binding of cations to a negatively charged membrane surface should neutralize the surface charge of that membrane [68]. By filtering a polyelectrolyte salt solution through the membranes, the intrinsic repulsion between similarly charged polyelectrolytes could be influenced via a nonsymmetrical 1:2 salt ratio. This would allow for the unequal neutralization of the oppositely charged solutions by the counter ion [69]. Using 1 mM and 5 mM salt solutions afforded this, as observed in Figure 12A–F. For NaCl salt, the flux reduced with 0 secs evaporation time, producing the highest flux, and 120 secs evaporation time, producing the lowest flux for both PSf/NMP and SPEEK-PSf/NMP membranes, as shown in Figure 12A,B.

**Figure 13:**
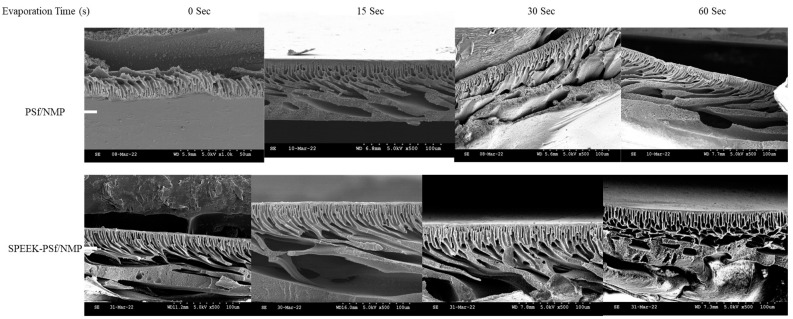
SEM cross-sectional images of PSf/NMP and 5% SPEEK-PSf/NMP membranes at various evaporation times. The evaporation time increases from the left to the right. The control/pristine membranes (PSf/NMP) membranes are at the top, while the membranes incorporated with SPEEK are at the bottom. All images were obtained at 5.0 kV, X500, 100 μm.

On the other hand, KCl and CaCl_2_ salts had the opposite effect, as shown in Figure 12C–F. The longer evaporation time led to higher fluxes compared to the lower evaporation times for both membranes. This could be attributed to the ionic radii of the repulsed species. While both Na^+^ and K^+^ are monovalent ions, they can form ion bridges that allow for increased flux. However, CaCl_2_ is divalent and thus has a larger attraction with much larger ionic radii, hence not forming a bridge [70]. SEM images did not show a difference in the pore structure’s morphology as the evaporation time was changed, as observed in Figure 13. A thicker selective layer was observed in the PSf/NMP membrane when the evaporation time was increased, but not with 5% SPEEK-PSf/NMP membranes. To allow for high flux and maximum contact with the negative surface charge, 60 s was chosen for the evaporation time.

#### 3.3.2. Effect of Charge Repulsion

**Figure 14:**
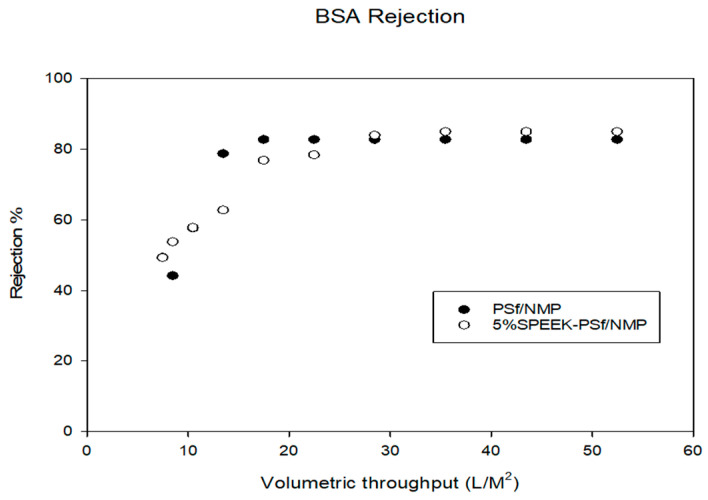
The percentage of BSA proteins rejected by both PSf/NMP and 5% SPEEK-PSf/NMP membranes. This was evaluated using UV-Vis spectrometry.

To understand how the introduction of the negative charges on the membrane surfaces affected its performance, BSA proteins were filtered through the membranes because BSA has been extensively studied in the literature for the evaluation of polymeric membrane performance [71] due to its inert behavior with respect to biochemical reactions, low cost, and handling ease [72]. With a size of 65 kDa, BSA was larger than the MWCO of the membranes (Figure 7), so it would be expected to be completely rejected. However, due to pore tortuosity and membrane compaction under pressure, the passage of BSA was observed [73,74]. The results showed that after ten filtrations, 5% SPEEK-PSf/NMP membranes had larger overall flux values compared to PSf/NMP membranes. Protein rejection (Figure 14) showed that the rejection of BSA when the 5% SPEEK polymer was incorporated averaged 84% rejection after the 10th filtration, while it was 80% for PSf/NMP membranes; however, these are not significantly different. The hydrophobic BSA protein would have a greater potential to adsorb on PSf/NMP membranes with a contact angle of 75 ± 0.5° compared to the 5% SPEEK-PSf/NMP membranes with a contact angle of 67 ± 1.1°, as previously stated (Figure 9), leading to increased fouling and hence limiting the performance of the membrane.

#### 3.3.3. Effects of Applied Voltage Potential

**Figure 15:**
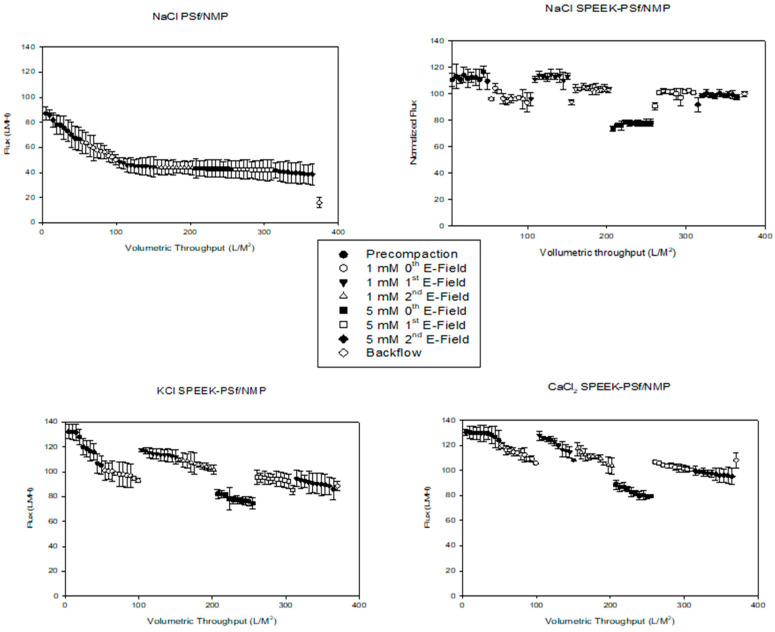
Graphs showing the effects of the applied potential on the performance of the PSf/NMP on the top left 5% SPEEK-PSf/NMP membranes (**top right**), using NaCl salt, and for KCl and CaCl_2_ salts on the (**bottom left**) and (**bottom right**), respectively. In both cases, pre-compaction with water is carried out before membrane filtration. This shows the voltage dependence where flux measurements were taken with no voltage applied (0th E-field), when forward voltage was applied (1st E-field), and when reverse voltage was applied (2nd E-field), as well as the salt concentration dependence where 1- and 5-mM salt solutions are used at 30 psi operating pressures.

When voltage was applied to the surface of the membrane, the permeability of the membrane increased at both low and high salt concentrations, as shown in Figure 15. The addition of the charge can have a significant impact on the membrane’s separations properties (ionic conductivity and mechanical property), as the addition of a charge can modify the PSf’s crystallinity, the porosity of the membrane, and the wettability of the composite separator [75,76]. Theoretically, an electrolyte solution that comes into contact with a surface should generate a charge on the surface of the membrane, which can then redistribute the ions in the electrolyte solution. This allows the counter ions to be attracted to the membrane surface and the co-ions to be repelled [77]. The concentration of counter ions with an opposite Cl^−^ sign is higher on the surface of the membrane than in the bulk solution, consequentially making the concentration of the co-ions smaller on the membrane surface. This creates a potential difference at the interface. Applying the supplied voltage in an electric field where the co-ions have the same charge as the membrane’s surface results in increased ion repulsion due to the increased forces between the two objects [78,79].

As observed in Figure 15, the pre-compaction of the 5% SPEEK-PSf/NMP membranes produced flux values that were on average above 100 LMH. Passing through an electrolyte solution of NaCl at 1 mM reduced the flux of the 5% SPEEK-PSf/NMP membranes by approximately 10%. The introduction of the first E-field increased the flux back to their pre-compaction levels. This could have been caused by the increased repulsion of charges brought about by the supplied electric field. The second E-field where the voltage was supplied in the opposite direction experienced a reduction in the flux; however, the flux was still higher than that without the voltage applied. Increasing the concentration of the electrolyte solution from 1 mM to 5 mM led to a reduction in the flux of the membranes, as expected. There was also no difference in the flux between the first and second E-field when a 5 mM salt solution was used. In general, changing the concentration of the feed directly influences sorption at the liquid/membrane interface. Since diffusion in the membrane is concentration-dependent, the water flux generally decreases with increasing salt concentrations in the feed due to increased transport resistance in the liquid boundary layer and concentration polarization [80]. The results showed that when voltage was applied across the surface of a negatively charged membrane, flux was independent of the external salt concentration. For PSf/NMP membranes, there was no effect on the flux of the membrane in the presence of the applied potential, and their overall flux values were less than those of the 5% SPEEK-PSf/NMP membranes.

Backflow was carried out to evaluate the potential for the reuse of the membrane after salt filtration. The results showed that after washing, the membrane flux was restored back to pre-compaction levels for the 5% SPEEK-PSf/NMP membranes. This indicated that the 5% SPEEK-PSf/NMP membranes had the potential to be reused. On the other hand, the backwash of PSf/NMP membranes did not improve the flux. This indicated that compared to the 5% SPEEK-PSf/NMP membranes, the PSf/NMP membranes had less potential of being reused after salt filtration. Similar results were obtained for KCl and CaCl_2_ salts, which are also observed in Figure 15. This indicated that regardless of the ionic radii of the salt, an application of an external potential to the surface of the negatively charged 5% SPEEK-PSf/NMP membranes improved the performance of that membrane in the repulsion of the charged species.

## 4. Conclusions

Although there has been substantial research directed toward SPEEK and PSf as individual polymers for membrane formation, very few studies have focused on their composite morphological functional properties. In this paper, it was demonstrated that SPEEK-PSf/NMP membrane composites could be produced with increased hydrophilicity and higher negative surface charge via the incorporation of the 5% SPEEK polymer. Interestingly, this surface charge was greater in the 5% SPEEK membrane compared to its 10% counterpart, which was likely due to the incorporation of the SPEEK into the matrix for okthe 10% membranes, as suggested by XPS depth profiling. This outcome corresponded with the results of the NMR, water uptake, and IEC analysis. When compared to the 10% SPEEK-PSf/NMP membranes, the 5% SPEEK-PSf/NMP membranes had the highest SPEEK integration from NMR analysis, increased water uptake percentage, and improved ion exchange capacity. Due to this, 5% SPEEK-PSf/NMP membranes were utilized for filtration studies. In terms of flux/permeability performance, SPEEK-PSf/NMP membranes displayed consistently larger overall flux values compared to the PSf/NMP membranes. Furthermore, in the presence of applied voltage, the 5% SPEEK-PSf/NMP membranes showed the potential to reject charged species and behave as electro-dialytic membranes that are capable of being backwashed and reused.

## Figures and Tables

**Table 1:** Percentage of solvent and polymers present in each membrane. The membranes were synthesized via NIP processes, and NMP was the solvent utilized in each membrane.

Membrane Composition	Solvent (NMP) %	Total Polymer % (PSf + SPEEK)	% SPEEK within Polymer
PSf/NMP	83	17	0
5% SPEEK-PSf/NMP	83	17	5
10% SPEEK-PSf/NMP	83	17	10

**Table 2:** Various salts, NaCl, KCl, and CaCl_2_ along with the concentrations used in filtration studies. These studies were carried out in a dead-end filtration cell.

Feed Composition	Feed Concentration
NaCl	1 mM, 5 mM
KCl	1 mM, 5 mM
CaCl_2_	1 mM, 5 mM
BSA Protein	100 ppm

**Table 3:** XPS elemental composition of membranes made of PSF/NMP, 5% SPEEK-PSf/NMP, and 10% SPEEK-PSf/NMP.

	PSf/NMP		5%SPEEK-PSf/NMP		10%SPEEK-PSf/NMP	
Name	PeakBE	Atomic%	PeakBE	Atomic%	PeakBE	Atomic%
C1s	284.92	75.71	284.3	81.61	286.88	76.66
O1s	532.15	22.35	532	15.38	534.27	21.15
S2p	167.91	1.95	167.8	3.01	169.87	2.18

**Table 4:** IEC and SD measurements of membranes. IEC measurements were calculated straight from the graphs while the SD values were obtained from the IEC measurements.

Membrane	IEC	SD
PSf/NMP	0.200286	0.05922
5% SPEEK-PSf/NMP	0.608485	0.1842
10% SPEEK-PSf/NMP	0.4186	0.1247

## Data Availability

The data presented in this study are available upon request from the corresponding author.

## Supplementary Materials

The following supporting information can be downloaded at: https://www.mdpi.com/article/10.3390/membranes13070675/s1, Figure S1: arrow pushing mechanism for the sulfonation of PEEK; Figure S2: EDS spectra of PSf/NMP (B2) and 5% SPEEK-PSf/NMP; Figure S3: IEC graphs for PSf/NMP membranes (A), 5% SPEEK-PSf/NMP membranes (B), and 10% SPEEK-PSf/NMP membranes (C).
