# Supplementary material for: Effects of the Applied Potential on the Performance of Polysulfone Membranes Functionalized with Sulfonated Polyether Ether Ketone Polymers
Source: Membranes (Basel). 2023 Jul 18;13(7):675. doi: 10.3390/membranes13070675 (PMC10386516; doi:10.3390/membranes13070675)
Supplement: Supplementary file 1 [file membranes-13-00675-s001.zip › membranes-2478791-supplementary.pdf]

Supplementary data

PEEK Sulfonation:

Figure 1, bellow shows a simpplified arrow pushing mechanism for the sulfonation process of PEEK polymer. It is a 2nd order electrophilic substition reaction that places the sulfone functional group on the quinone backbone preferentailly.

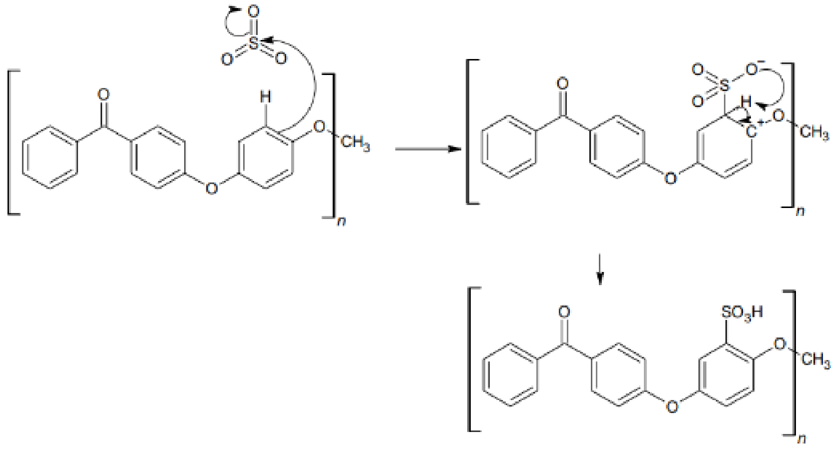

Figure S1 arrow pushing mechanism for the sulfonation of PEEK.

SEM-EDS

EDS spectra showed that the sulfur content percentage was double in the 5% SPEEK-PSf/NMP membranes as compared to those membranes without SPEEK incorporated in them. This agreed with data obtained via XPS analysis.

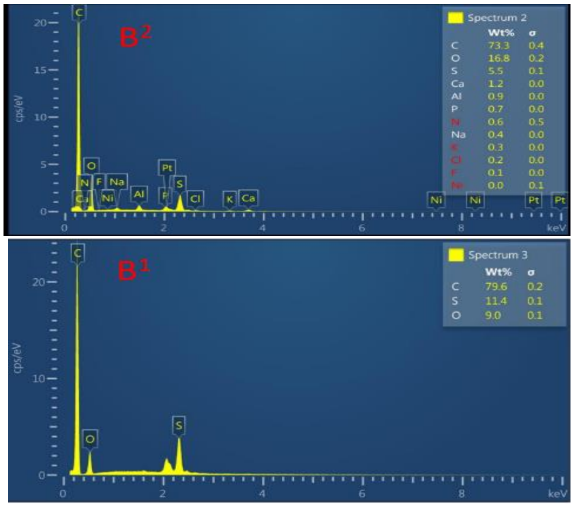

Figure S2 EDS spectra of PSf/NMP (B2) and 5% SPEEK-PSf/NMP

IEC graphs:

Figure S3 shows the IEC of the various membranes PSf/NMP (A), 5%SPEEK-PSf/NMP (B), and 10% SPEEK-PSf/NMP (C) over a range of pH. The line extrapolated at pH of 7 indicates the vollume of base it took to neutralize the acid. From the data, as the amount of SPEEK incoporated into the membrane increased, the more base was required for the acid to get neutralized.

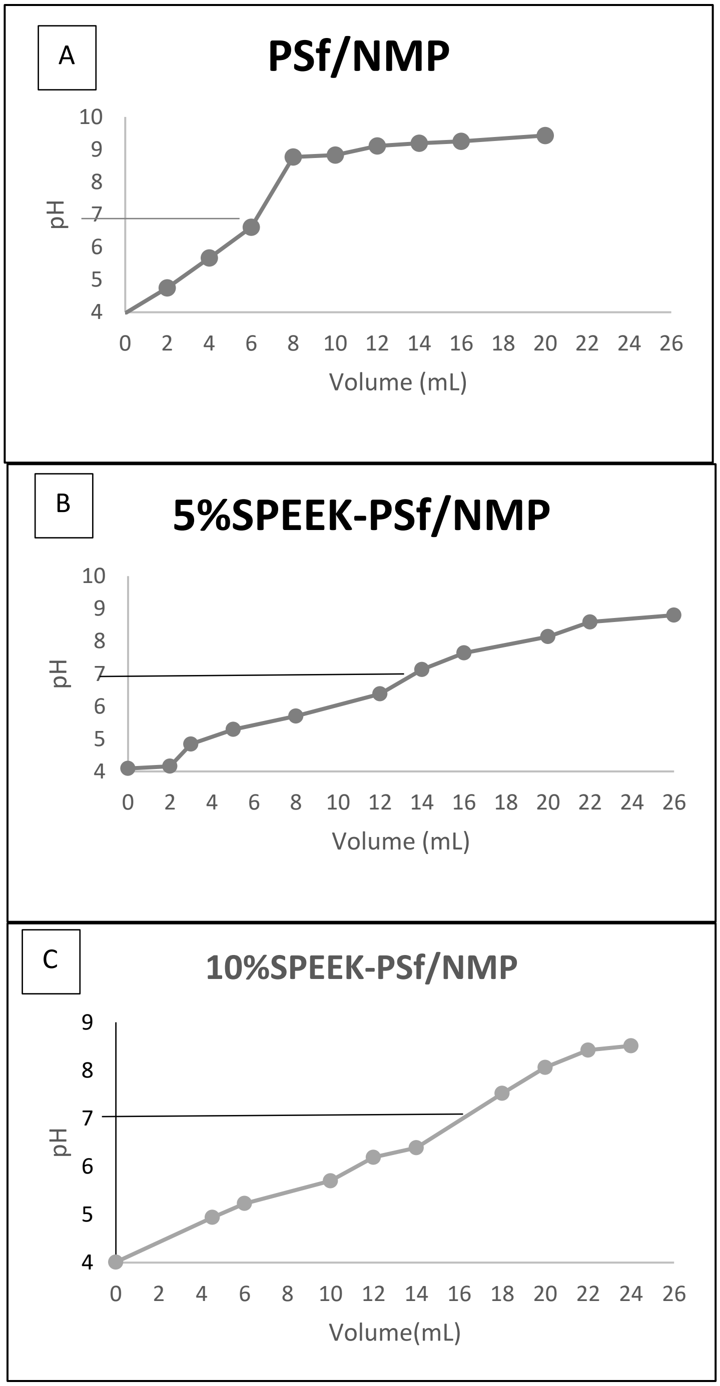

Figure S3 IEC graphs for PSf/NMP membranes (A), 5% SPEEK-PSf/NMP membranes (B), and 10% SPEEK- PSf/NMP membranes (C)
